# CD146^+^CAFs promote progression of endometrial cancer by inducing angiogenesis and vasculogenic mimicry via IL-10/JAK1/STAT3 pathway

**DOI:** 10.1186/s12964-024-01550-9

**Published:** 2024-03-08

**Authors:** Zhicheng Yu, Qian Zhang, Sitian Wei, Yang Zhang, Ting Zhou, Qi Zhang, Rui Shi, Dmitry Zinovkin, Zahidul Islam Pranjol, Jun Zhang, Hongbo Wang

**Affiliations:** 1 Department of Obstetrics and Gynecology, Union Hospital, Tongji Medical College, Huazhong University of Science and Technology, Wuhan, Hubei People’s Republic of China; 2 Department of Obstetrics and Gynecology, The First Affiliated Hospital of University of Science and Technology of China, Hefei, People’s Republic of China; 3 Department of Pathology, Gomel State Medical University, Gomel, Republic of Belarus; 4 Department of Biochemistry, School of Life Sciences, University of Sussex, Falmer, UK; 5 Clinical Research Center of Cancer Immunotherapy, Wuhan, Hubei People’s Republic of China

**Keywords:** Endometrial cancer, Cancer-associated fibroblast, Interleukin 10, Vasculogenic mimicry

## Abstract

Heterogeneous cancer-associated fibroblasts (CAFs) play important roles in cancer progression. However, the specific biological functions and regulatory mechanisms involved in endometrial cancer have yet to be elucidated. We aimed to explore the potential mechanisms of heterogeneous CAFs in promoting endometrial cancer progression. The presence of melanoma cell adhesion molecule (MCAM; CD146) positive CAFs was confirmed by tissue multi-immunofluorescence (mIF), and fluorescence activated cell sorting (FACS). The biological functions were determined by wound healing assays, tuber formation assays and cord formation assays. The effects of CD146^+^CAFs on endometrial cancer cells were studied in vitro and in vivo. The expression level of interleukin 10 (IL-10) was measured by quantitative real time polymerase chain reaction (qRT-PCR), western boltting and enzyme linked immunosorbent assays (ELISAs). In addition, the transcription factor STAT3 was identified by bioinformatics methods and chromatin immunoprecipitation (ChIP). A subtype of CAFs marked with CD146 was found in endometrial cancer and correlated with poor prognosis. CD146^+^CAFs promoted angiogenesis and vasculogenic mimicry (VM) in vitro. A xenograft tumour model also showed that CD146^+^CAFs can facilitate tumour progression. The expression of IL-10 was elevated in CD146^+^CAFs. IL-10 promoted epithelial-endothelial transformation (EET) and further VM formation in endometrial cancer cells via the janus kinase 1/signal transducer and activator of transcription 3 (JAK1/STAT3) signalling pathway. This process could be blocked by the JAK1/STAT3 inhibitor niclosamide. Mechanically, STAT3 can bind to the promoter of cadherin5 (CDH5) to promote its transcription which may be stimulated by IL-10. We concluded that CD146^+^CAFs could promote angiogenesis and VM formation via the IL-10/JAK1/STAT3 signalling pathway. These findings may lead to the identification of potential targets for antiangiogenic therapeutic strategies for endometrial cancers.

**Supplementary Information:**

The online version contains supplementary material available at 10.1186/s12964-024-01550-9.

## Introduction

As one of the most common gynecological cancers, the incidence of endometrial cancer is increasing [1]. While surgery is the main treatment strategy, part of advanced or recurrent patients are lack of effective treatment due to the poor sensitivity to chemotherapy or radiotherapy.. Antiangiogenic therapy has achieved certain results, but not all patients benefit from it [2, 3].

The growth and progression of malignant tumours can not be separated from abundant microcirculation to provide oxygen and nutrients. Angiogenesis and vasculogenic mimicry (VM) are both important types of blood supply for tumours and may lead to poor prognosis [4, 5]. According to the National Comprehensive Cancer Network (NCCN) guidelines, antiangiogenesis therapy is recommended for patients with advanced or recurrent endometrial cancer, but some patients exhibit undesirable therapeutic responses [6]. VM is a new tumour microcirculation pattern that is independent of endothelial cells [7]. Epithelial-endothelial transition (EET) is an essential process in the formation of VM characterized by downregulated expression of the epithelial marker E-cadherin and upregulated expression of the endothelial markers VE-cadherin and vimentin [8, 9]. VE-cadherin is a VM biomarker and plays a crucial role in the formation of VM [10]. Previous studies have shown that VM is correlated with aggressive phenotypes such as poorly differentiated, invasive and metastatic phenotypes, which indicates that VM might be a potential therapeutic target [11]. However, the existence and regulatory mechanisms of VM in endometrial cancer remain to be clarified.

The Tumour microenvironment (TME) is a complex ecosystem that is composed of various cells such as epithelial cells, stromal cells, immune cells and extracellular components [12]. The homeostasis of the microenvironment is a prerequisite for tumour growth and progression. Cancer-associated fibroblasts (CAFs) are the principal component of the TME and they play important roles in matrix remodelling, angiogenesis and immune regulation during tumour progression [13–15]. The functional diversity of CAFs is due to their genotype and molecular heterogeneity. Therefore, determining the specific biological functions of heterogeneous CAFs is important. We divided CAFs from endometrial cancer patients into four subtypes by single-cell sequencing, and one of the subtypes, named vCAFs (marked with CD146), was correlated with poor prognosis [16].

In this study, we demonstrated that CD146 was expressed on some CAFs, although this molecule was previously considered a specific marker of endothelial cells. We also showed that CD146^+^CAFs promoted the progression of endometrial cancer by inducing angiogenesis and VM via the IL-10/JAK1/STAT3 pathway in vitro and in vivo exploration.

## Materials and methods

### Collection of patient samples

All endometrial cancer (*n* = 10) and normal endometrium (*n* = 5) samples were collected from Union Hospital, Tongji Medical College, Huazhong University of Science and Technology approved by the Medical Ethics Committee of Tongji Medical College, Huazhong University of Science and Technology (No. 2020-S218) and patients enrolled in the study provided written informed consent. No patients received treatment prior to surgery, such as chemotherapy or radiotherapy. The control normal endometrium was obtained from patients who underwent hysterectomy due to nonmalignant gynecological diseases.

### Tissue multi-immunofluorescence

Tissue multi-immunofluorescence was done to validate the expression of EPCAM, α-SMA and CD146 in endometrial cancer. The tissue sections were deparaffinized, hydrated and rehydrated according to the standard protocols. Antigen retrieval was performed by high-temperature heating (EDTA, pH9.0) for ninety seconds. Endogenous peroxidase and non-specific binding sites were blocked by 10% serum consistent with secondary antibodies for thirty minutes in 37℃. The sections were incubated with rabbit monoclonal anti-EPCAM (1:500, Abcam, UK, Cat: ab223582) overnight at 4℃ and the secondary antibody was incubated with the samples for 45 min at 37℃. Tyramide signal amplification (TSA) stain was carried out as standard. Then the sections were repeated the above steps while different antibodies (Rabbit monoclonal anti-α-SMA, 1:500, Abcam, UK, Cat: ab7817, Rabbit monoclonal anti-CD146, 1:250, Abcam, UK, Cat: ab75769) were used. DAPI was used to stain the nucleus in dark conditions.

### Isolation of primary fibroblasts and cell culture

The fresh excised tissue samples were collected under sterile conditions. Then the tissue was cut into small pieces and digest it in cell incubator with collagenase I (2 mg/ml, Sigma, USA, Cat: 1148089) for 1 h [17]. The digestion was terminated by DMEM (Gibco) medium containing 10% fetal bovine serum (FBS, HyClone). Cell suspension was filtered by 70μm and 40μm strainer respectively. Then the cells were washed and cultured in DMEM/F12 media supplemented with 10% FBS and 1% penicillin/streptomycin at 37℃. All the primary fibroblasts used in this study were less than 8 passages. HUVEC and endometrial cancer cell lines ishikawa and KLE were purchased from Zhong Qiao Xin Zhou Biotechnology (Shanghai, China) and cultured in DMEM/F12 supplemented with 10% FBS.

### Cell immunofluorescence (IF)

IF was used to character the classic markers of CAFs. Cells were digested and implanted on cover glass beforehand. After washed three times using PBS, 10% formaldehyde solution fixed. The cover glass was incubated with rabbit monoclonal anti-α-SMA (1:500, Abcam, UK, Cat: ab7817) and rabbit monoclonal anti-FAP (1:500, Abcam, UK, Cat: ab314456) overnight at 4℃. Then the secondary antibody was incubated with the samples for 45 min at 37℃ in dark conditions. DAPI was used to stain the nucleus for 10 min.

### Fluorescence Activated Cell Sorter (FACS)

CAFs were processed to cell suspension and counted. The antibodies were added as manufacturer’s guidance (FITC anti-human CD105 Antibody, Biolegend, USA, Cat: 323203. FITC anti-human CD90 Antibody, Biolegend, USA, Cat: 328107. PE anti-human CD146 Antibody, Biolegend, USA, Cat: 361005. PE anti-human CD34 Antibody, Biolegend, USA, Cat: 378603. PE anti-human HLA-DR Antibody, Biolegend, USA, Cat: 327007. PE anti-human CD73 Antibody, Biolegend, USA, Cat: 344003). After incubated for 30 min at 37℃ in dark conditions, the samples were analyzed or sorted. Then the cells were collected and cultured in DMEM/F12 media supplemented with 10% FBS and 1% penicillin/streptomycin.

### Collection of conditioned media

CAFs/NFs were washed with PBS twice and cultured in media with serum-free DMEM/F12 for the subsequent 24 h. Conditioned medium was collected and centrifuged at 1000g for 15 min, and the supernatant was concentrated with Centricon YM3 filters (Milipore, 0.22μm, USA).

### Wound healing assay

HUVEC were digested and implanted in 6-well plates. After the cells overspread the well, three straight lines were performed using micropipette tips. PBS was used to remove the floating cells. Then the serum-free conditioned media were added respectively and the area of scratch were recorded after scratch and 6 h later. Each experiment was performed in triplicate.

### Tuber formation assay

The matrigel (BD356234, USA) was thawed at 4℃ and added to each well of a 24-well plate (250μl/well). The plate was put into cell incubator to solidify the matrigel. HUVEC was re-suspended in different conditioned media and the cell suspension was seeded with 1 × 10^5^ cells per well. The tube formation was observed after incubating 6–8 h. Each experiment was performed in triplicate.

### Cord formation assay

The matrigel (BD356234, USA) was thawed at 4℃ and added to each well of a 24-well plate (250μl/well). The plate was put into cell incubator to solidify the matrigel. Endometrial cancer cell lines were re-suspended in different conditioned media and the cell suspension was seeded with 1 × 10^5^ cells per well. The tube-like structure was observed after incubating 24 h. Each experiment was performed in triplicate.

### Western blot

Cells were lysed using RIPA with 1 mmol/L PMSF and a protease Cocktail Inhibitor at 4℃ for 30 min. After centrifugation at 12,000 rpm for 15 min, the supernatant was harvested and the protein concentration was measured using a BCA Assay Kit. Whole cell lysates were fractionated by SDS-PAGE and transferred onto polyvinylidene difluoride (PVDF) membranes. Blots were blocked with 5% skimmed milk powder and incubated with antibodies targeting IL-10 (1:1000, Proteintech, China, Cat: 60269–1-Ig), VE-Cadherin (1:1000, Proteintech, China, Cat: 66804–1-Ig), E-Cadherin (1:2000, Proteintech, China, Cat: 20874–1-AP), Vimentin (1:1000, Proteintech, China, Cat: 10366–1-AP), JAK1 (1:1000, Cell signaling technology, USA, Cat: 3344), p-JAK1 (1:1000, Abclone, China, Cat: AP0530), STAT3 (1:1000, Abclone, China, Cat: A19566), p-STAT3 (1:1000, Cell signaling technology, USA, Cat: 9145), GAPDH (1:10000, Proteintech, China, Cat: 60004–1-Ig). After incubation with HRP-conjugated secondary antibodies (1:8,000, Affinity, USA, Cat: S0001, S0002), the blots were detected using enhanced chemiluminescence detection reagents on Image Lab Software.

### RNA extraction and quantitative real time polymerase chain reaction (qRT-PCR)

Total RNA was isolated from samples of cells using TRizol following the manufacturer’s instruction, cDNA was prepared using a PrimeScriptRT Reagent Kit. qRT-PCR were carried out by the Biosystem StepOne Plus PCR System and Real-time PCR Kit with specific primers. The sequence of primes was exhibited as Supplementary Table 1. The expression level of mRNA was calculated relative to GAPDH expression using the 2^−ΔΔCt^ method.

### Enzyme linked immunosorbent assay (ELISA)

ELISA was conducted by RUIXIN Biotech IL-10 ELISA KIT (Quanzhou, China, Cat: RX103064H). The conditioned media and standard samples were prepared in advanced and added to each reaction well as the manufacturer’s instruction. After incubate for 1 h, the optical density (OD) value at 450nm of each well was measured by microplate reader. The concentration of protein in the conditioned media was calculated through standard curve.

### Half maximal inhibitory concentration (IC50)

IC50 of niclosamide was measured by Cell Count Kit 8 (CCK8, Targetmol, China) following the manufacturer’s instructions. Briefly, 1 × 10^4^ endometrial cancer cells in a 96-well plate were treated with different indicated concentrations of niclosamide and incubated at 37℃ overnight. Then, 10µl of CCK-8 solution was added into each well. Cells were then incubated for 1 h. The OD value at 450nm was measured by microplate reader.

### Chromatin immunoprecipitation assay (CHIP)

CHIP was performed using Ishikawa cells which were treated with IL-10 in advance. Then we added 37% formaldehyde at 1% final concentration to the cells for 10 min at room temperature to cross-link proteins to DNA. After that, the remaining steps were carried out according to the manufacturer’s instructions for the ChIP assay kit (Beyotime, China, Cat: P2078). The antibodies used were anti-p-STAT3 (CST, USA, Cat: 9145) and normal rabbit IgG (Proteintech, China, Cat: B900610). The enriched DNA was analyzed utilizing the primers by real-time PCR.

### Nude mice xenograft assays

Female BALB/c nude mice (4–5 weeks) were purchased from China Charles River and housed in a standard pathogen-free environment laboratory. The mice were randomized into four groups. Ishikawa, Ishikawa + NFs, Ishikawa + CD146^−^CAFs, Ishikawa + CD146^+^CAFs were suspended in 100μl serum-free DMEM/F12 respectively and subsequently injected into the right flanks of the mice. After 21 days, the mice were cervical dislocated under anesthesia, and the weight, length, and width of xenografts were measured. Tumor volume = (length * width^2^)/2. Tumor samples were partially embedded in paraffin for histopathological analysis. Animal experiments were performed according to the protocols approved by Tongji Medical College’s Animal Care and Use Committee (No. 2022-S3018).

### Immunohistochemistry (IHC)

The sections were incubated with E-Cadherin, Ve-Cadherin and vimentin antibody respectively (1:500, Proteintech, China, Cat: 20874–1-AP, 66804–1-Ig, 10366–1-AP). After incubated at 4 ℃ overnights, the stained sections were incubated with secondary antibody at room temperature for 30 min in darkness and visualized with DAB solution. Finally, the nuclei were counterstained by Mayer’s haematoxylin. A Motic microscope (Motic, China) was used to visualize and photograph the sections. The IHC staining scores were evaluated by two independent observers blinded to the corresponding patients based on the staining intensity (SI) and the percentage of immunoreactive cells (PR) [18]. The SI score was calculated from 0 to 3: 0 = no staining; 1 = weak staining; 2 = moderate staining; and 3 = strong staining. The PR was scored from 1 to 4: 1 = 0–25%; 2 = 26–50%; 3 = 51–75%; and 4 = 75–100%. The PR and the SI were multiplied to produce a weighted score for each patient. A score of 8–12 was defined as a high expression level and a score of 0–7 was defined as low expression.

### CD31/PAS double staining

IHC staining with CD31 (1:300, Proteintech, China, Cat: 66065–2-Ig) was performed on the sections as described above prior to PAS staining. Then, the slides were treated with periodic acid solution (PAS) for 10 min and washed with distilled water for 5 min. In a dark chamber, the slides were submerged in Schiff solution for 15 min at 37℃. The criteria used were as follows: 1. absence of vascular endothelial cells on the inner wall of the VM blood vessel, 2. vascular-like channels are lined with tumor cells, 3. positive for PAS staining but negative for CD31 staining.

### Statistical analysis

The experimental data of every 3 independent replicates were performed as the mean ± standard deviation (SD). The Tukey’s test was used for comparisons between two independent sample groups. One-way ANOVA or two-way ANOVA tests were used for comparisons between multiple groups. *P* < 0.05 was considered as statistically significant difference. * *P* < 0.05, ** *P* < 0.01, *** *P* < 0.001. GraphPad Prism 8 (GraphPad Software, USA) was used for statistical analysis.

## Results

### Identification of CD146^+^CAFs in endometrial cancer

**Fig. 1:**
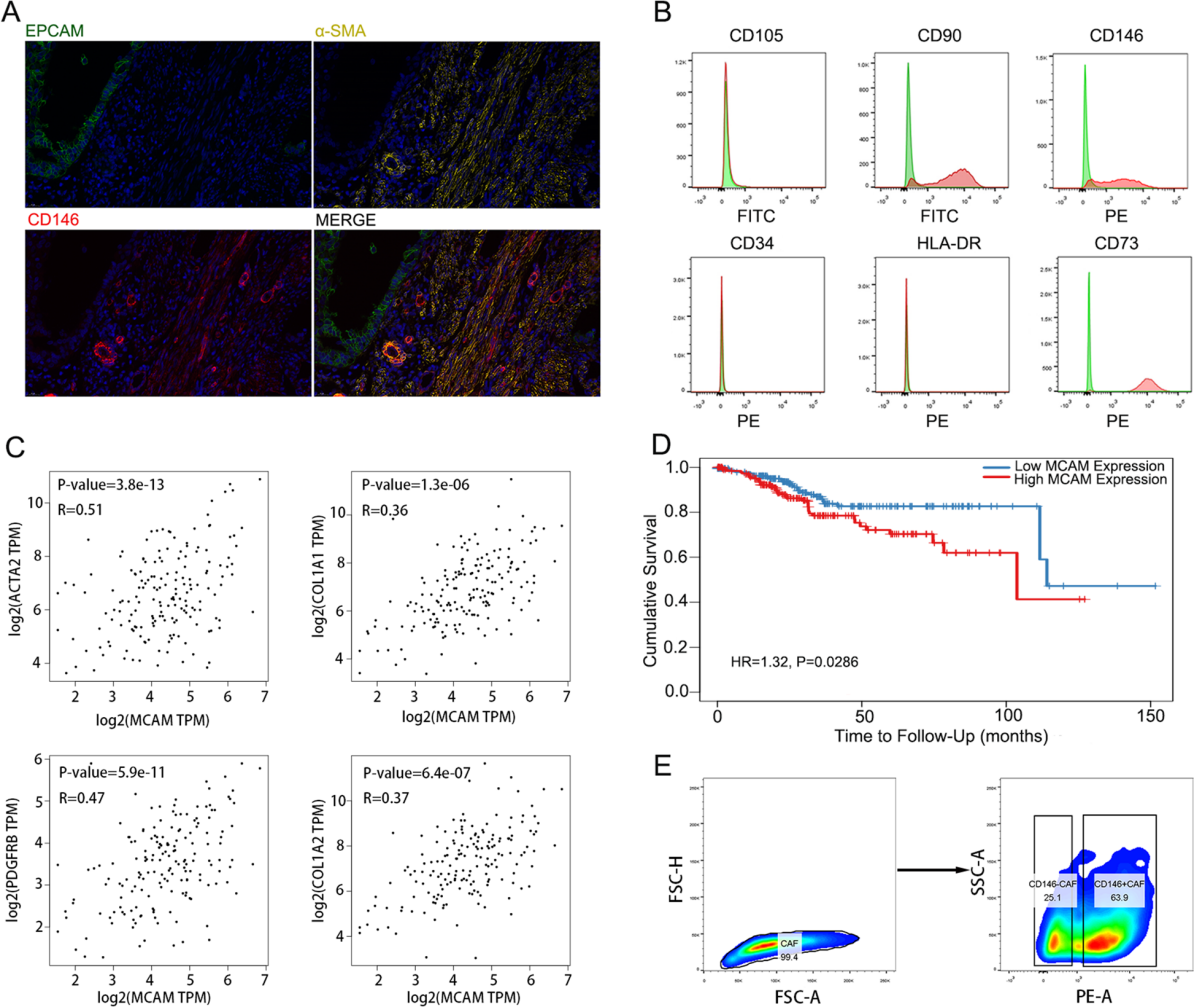
Identification of CD146^+^CAFs in endometrial cancers. **A** IF staining of EPCAM, α-SMA and CD146 in endometrial cancer; **B** Flow cytometry analysis of the expression of CD105, CD90, CD146, CD34, HLA-DR and CD73 in endometrial cancer; **C** Correlation analysis of MCAM with ACTA2, COL1A1, PDGFRB and COL1A2 in endometrial cancers by Gene Expression Profile Interactive Analysis (GEPIA; http://gepia.cancer-pku.cn); **D** Prognosis significance of MCAM in endometrial cancer by The Human Protein Atlas (https://www.proteinatlas.org/); **E** Sorting CD146 positive and negative CAFs by FACS

To verify the presence of CD146^+^CAFs in endometrial cancer, We performed mIF assays aimed at evaluating EpCAM (epithelial marker), α-SMA (stromal marker) and CD146. The merged image shows that there were a group of CD146 positive CAFs in the interstitial region apart from the endothelial cells (Fig. 1A). We also isolated the fibroblasts from fresh excised endometrial cancer tissues and normal endometrium. IF staining for α-SMA and FAP demonstrated that fibroblasts derived from endometrial cancer samples were activated (Figure S1). Positive (CD73 and CD90) and negative (CD105, CD34 and HLA-DR) expression of markers in CAFs were also detected by flow cytometry (Fig. 1B). A group of CD146 positive CAFs was consistently identified in endometrial cancer (Fig. 1B). Moreover, the mRNA expression level of CD146 was positively correlated with that of classic markers of CAFs such as ACTA2, COL1A1, PDGFRB and COL1A2 (Fig. 1C). Patients with high CD146 expression had a relatively poor prognosis (Fig. 1D). These results indicated that a subtype of CAFs marked with CD146 was present in endometrial cancer which may induce its malignant progression. To investigate this phenomenon, we isolated the CD146 positive and CD146 negative CAFs for subsequent experiments through FACS to explore their biological functions.

### CD146^+^CAFs promote angiogenesis and vasculogenic mimicry in vitro

**Fig. 2:**
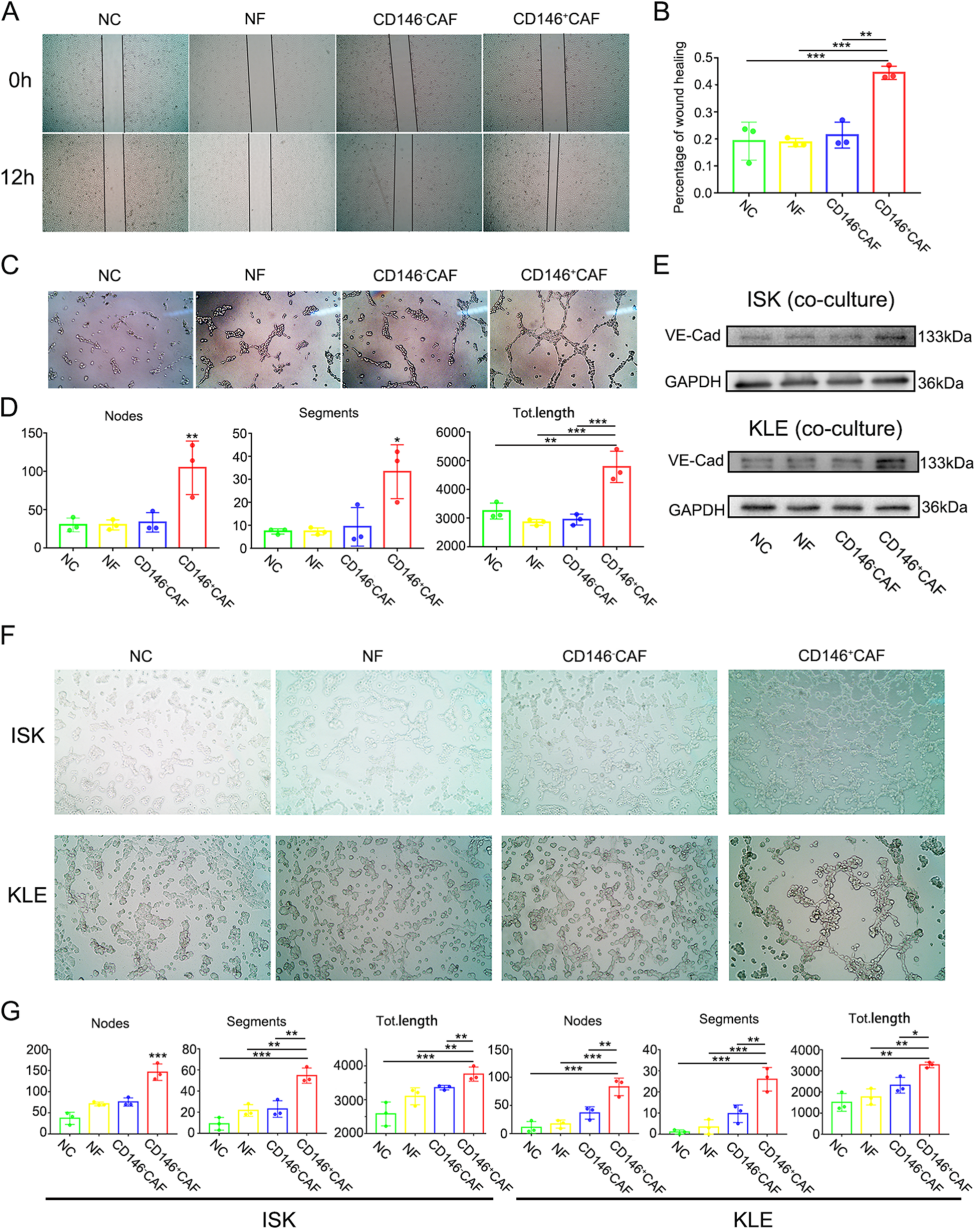
CD146^+^CAFs promote angiogenesis and VM in vitro. **A** Wound healing assays of HUVEC treated with different condition media; **B** The statistical results of wound healing assays; **C** Tube formation assays of HUVEC treated with different condition media; **D** The statistical results of tube formation assays; **E** The protein expression of VE-Cadherin in Ishikawa and KLE after treated with different condition media; **F** Cord formation assays of Ishikawa and KLE after treated with different condition media; **G** The statistical results of cord formation assays

Our previous research revealed that the differentially expressed genes enriched in CD146^+^CAFs were involved in the regulation of vasculature development, regulation of endothelial cell migration and regulation of angiogenesis et al. Therefore, we aimed to explore the proangiogenic characteristics of CD146^+^CAFs. The conditioned medium from CD146^+^CAFs promoted the migration and tube formation abilities of HUVECs in vitro compared with those from NFs or CD146^−^CAFs (Fig. 2A-C). The number of nodes and segments and the total length were significantly increased after cocultured with HUVECs (Fig. 2D). The mechanism of vasculogenic mimicry (VM), an important tumour microcirculation pattern, remains to be determined. The expression of the key molecule of VM, VE-Cadherin, was increased in endometrial cancer cells after treatment with conditioned medium from CD146^+^CAFs (Fig. 2E). Cord formation assays were performed as previously described to explore the VM formation ability. We concluded that CD146^+^CAFs significantly promote VM formation after cocultured for 24 h (Fig. 2F, G).

### CD146^+^CAFs upregulate IL-10 to facilitate vasculogenic mimicry

**Fig. 3:**
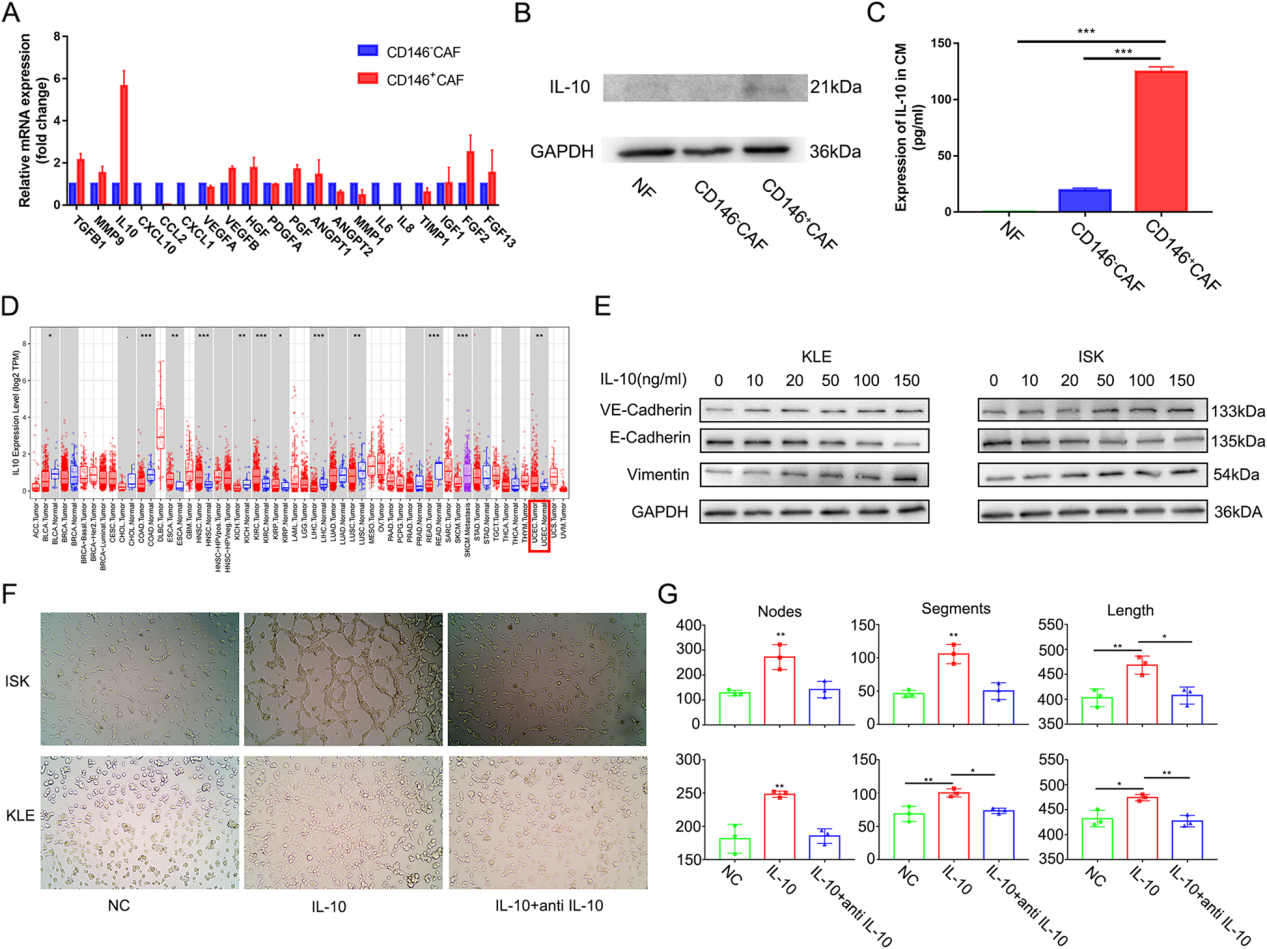
CD146^+^CAFs up-regulate IL-10 to facilitate VM. **A** PCR analysis of angiogensis related cytokines; **B** IL-10 protein expression in NF, CD146^-^CAFs and CD146^+^CAFs by western blot; **C** IL-10 expression in NF, CD146^-^CAFs and CD146^+^CAFs supernatant by ELISA; **D** The mRNA expression of IL-10 in cancers by Tumor Immune Estimation Resource (TIMER; https://cistrome.shinyapps.io/timer); **E** The protein expression of VE-Cadherin, E-Cadherin and Vimentin in Ishikawa and KLE after treated with concentration gradient IL-10; **F** Cord formation assays of Ishikawa and KLE after treated with IL-10 and IL-10 antibody; **G** The statistical results of cord formation assays

Cytokines play an important role in intercellular communication. Therefore, we detected the transcriptional levels of vascular related cytokines in CD146^+^CAFs and CD146^−^CAFs (Fig. 3A). Among them, IL-10 was elevated in CD146^+^CAFs. The expression levels of IL-10 in the protein and cell supernatants also increased significantly in CD146^+^CAFs, consistent with the increase in mRNA expression (Fig. 3B, C). Furthermore, the IL-10 expression level was significantly correlated with the MCAM (also named CD146) level (Figure S2A). Previous studies have suggested that IL-10 is a pleiotropic cytokine [19, 20]. This molecule is upregulated in many malignant tumours such as ESCA, HNSC and KIRC (Fig. 3D). In addition, compared with that in the normal endometrium, the expression of IL-10 in endometrial cancer was increased, indicating that it may be a tumorigenic cytokine in endometrial cancer (Figs. 3D and S2B). Survival analysis also revealed that patients with higher IL-10 expression had a worse prognosis (Figure S2C).

**Fig. 4:**
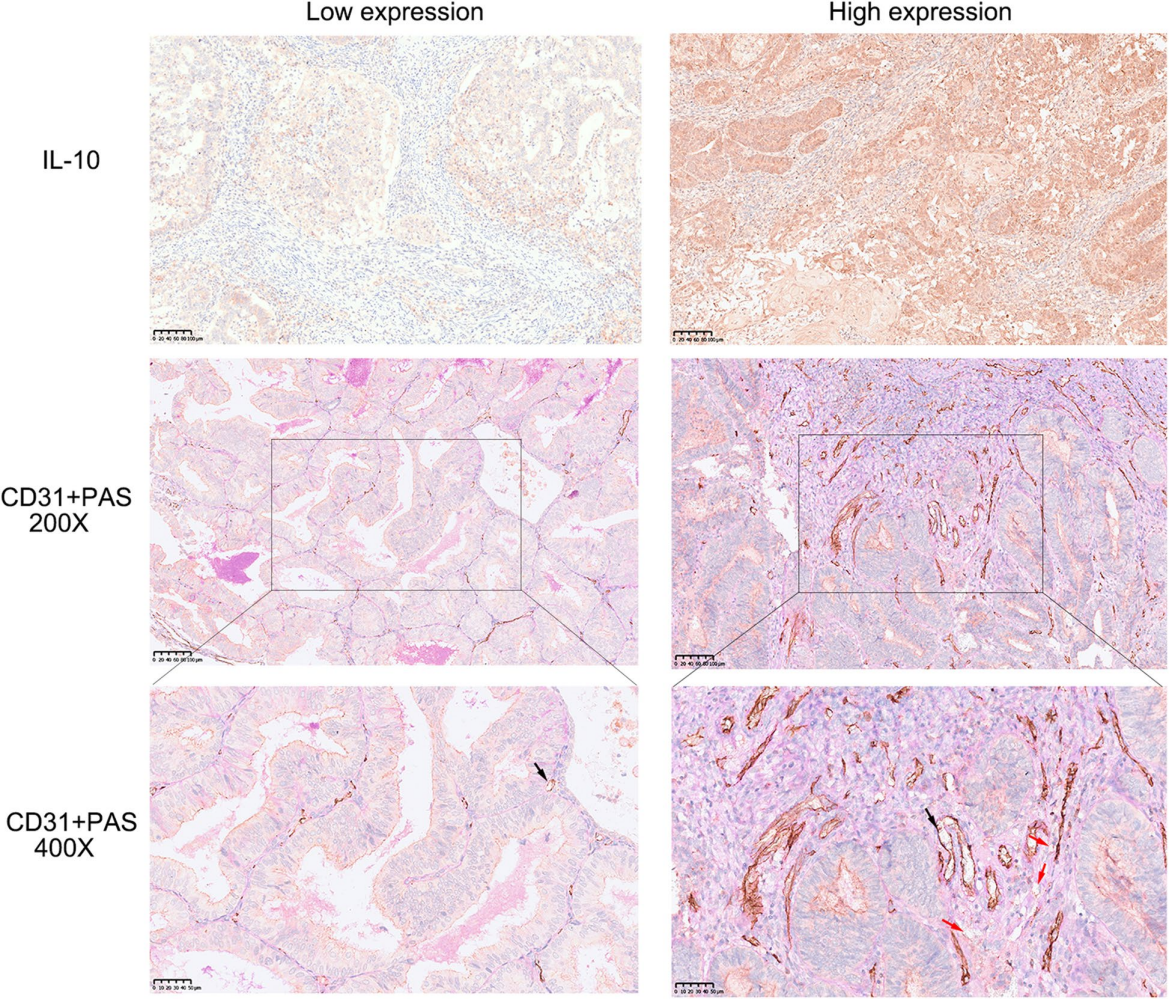
The expression of IL-10 and VM in endometrial cancer tissues (Red arrows represent for VM and black arrows represent for vessels)

We subsequently explored the expression of the IL-10 receptors IL-10RA and IL-10RB in endometrial cancer cell lines. Ishikawa and KLE cells were obtained and selected for subsequent research (Figure S2D). Epithelial-endothelial transition (EET) is an essential process in the formation of VM. Western blot analysis revealed that IL-10 promoted EET in endometrial cancer cells in a concentration-dependent manner (Fig. 3E). A cord formation assay was also performed and the number of nodes and segments and the total length were significantly increased after the addition of IL-10, and this effect could be blocked with an IL-10 neutralizing antibody (Fig. 3F, G). We also examined the relationships between IL-10 and VM in clinical samples. The numbers of VM were significantly greater in specimens from patients with high IL-10 expression than in those from patients with low IL-10 expression, indicating that IL-10 may stimulate the formation of VM in endometrial cancer (Figs. 4 and S2E).

### IL-10 induces VM via the JAK1/STAT3 pathway

**Fig. 5:**
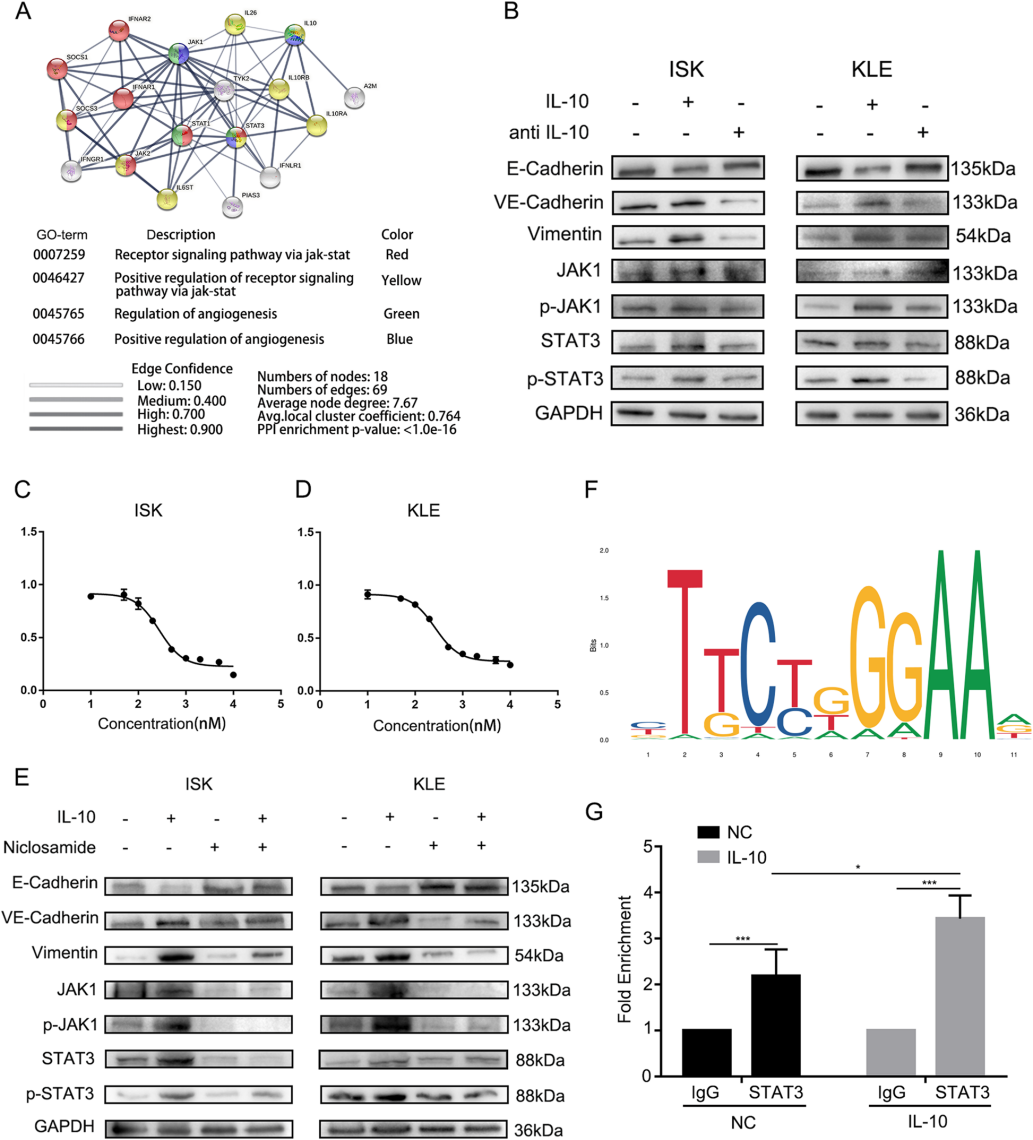
IL-10 enhance VM via JAK1/STAT3 pathway. **A** IL-10 associated networks predicted by STING (https://cn.string-db.org/); **B** The protein expression of VE-Cadherin, E-Cadherin, Vimentin and JAK1/STAT3 pathway in Ishhikawa and KLE after treated with IL-10 and IL-10 antibody; **C** The growth curve of Ishikawa after treated with concentration gradient niclosamide; **D** The growth curve of KLE after treated with concentration gradient niclosamide; **E** The protein expression of VE-Cadherin, E-Cadherin, Vimentin and JAK1/STAT3 pathway in Ishhikawa and KLE after treated with IL-10 and niclosamide; **F** The binding sites of STAT3 and CDH5 were predicted by the JASPAR database (https://jaspar.genereg.net/#). **G** ChIP assay verified that STAT3 could bind to CDH5 promoter in Ishikawa cells which could be stimulated by IL-10

STING (https://cn.string-db.org/) was used to explore IL-10 associated networks. The JAK1/STAT3 signalling pathway and regulation of angiogenesis were enriched for IL-10 (Fig. 5A). Then, IL-10 was blocked by a neutralizing antibody, and the phosphorylation of JAK1 and STAT3 in response to IL-10 was significantly decreased (Fig. 5B). Furthermore, the inhibition of the JAK1/STAT3 signalling pathway was also assessed by using a JAK1/STAT3 inhibitor (niclosamide 274.3nM for Ishikawa cells and 263.9nM for KLE cells) (Fig. 5C, D). Changes in VM biomarkers (E-Cadherin, VE-Cadherin, Vimentin) and the JAK1/STAT3 signalling pathway were detected. We found that the inhibition of JAK1/STAT3 downregulated VE-Cadherin, Vimentin and upregulated E-Cadherin (Fig. 5E). In addition, the VM formation was decreased after treatment with niclosamide (Figure S3A). These results indicate that the JAK1/STAT3 signalling pathways activated by IL-10 play a critical role in these morphological changes, leading to VM formation.

To determine the intrinsic mechanism of VM formation, we investigated the relationship between JAK1/STAT3 and the key VM molecule VE-Cadherin. The mRNA level of CDH5 was significantly correlated with JAK1 and STAT3 levels in endometrial cancer (Figure S3B). Subsequently, the specific STAT3 binding sites on the CDH5 promoter were also predicted by JASPAR (Fig. 5F). According to the predicted binding sites, we designed ChIP-qPCR primers and performed a ChIP assay. The results indicated that STAT3 was significantly enriched in the CDH5 promoter in Ishikawa cells and that this enrichment was further increased by IL-10 (Fig. 5G). DNA gel electrophoresis revealed that the length of the qPCR product was approximately 100–200 bp (Figure S3C). Taken together, these findings suggest that STAT3 can directly activate CDH5 transcription by binding to its promoter region in endometrial carcinoma and that IL-10 can further promote transcription.

### CD146^+^CAFs promote the progression of endometrial cancer by inducing angiogenesis and VM

**Fig. 6:**
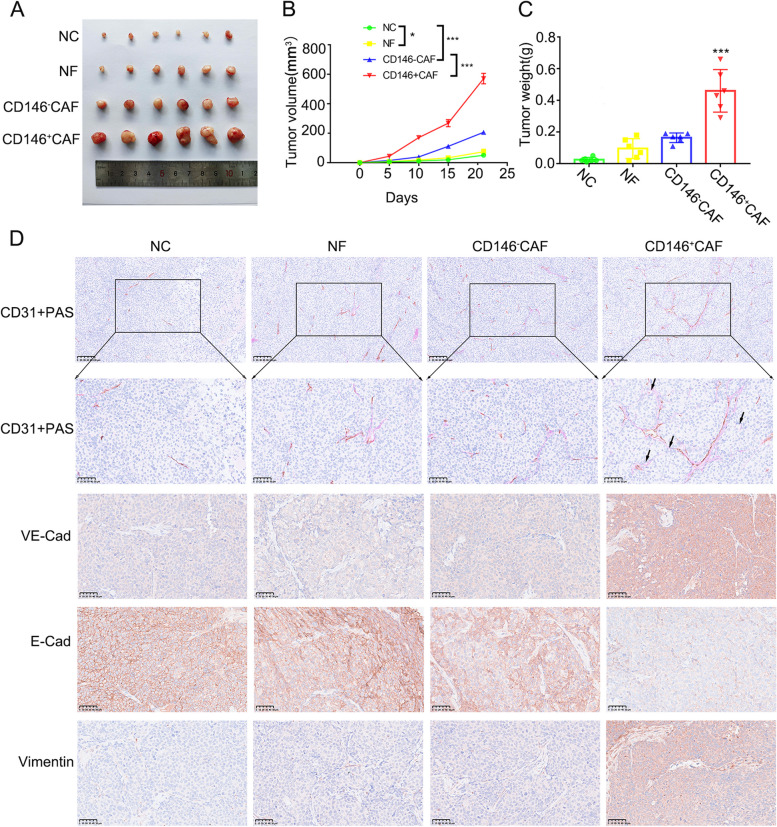
CD146^+^CAFs promote progression of endometrial cancer by inducing angiogenesis and VM. **A** Images of xenograft tumors from BALB/c-nude mice 21 days after the subcutaneous injection of cells in different groups; **B** Tumor volume according to the formula: (length * width^2^)/2; **C** Tumor weight of nude mice accessed on day 21; **E** PAS and CD31 IHC staining and VE-Cadherin, E-Cadherin, Vimentin IHC staining for xenograft tumors

**Fig. 7:**
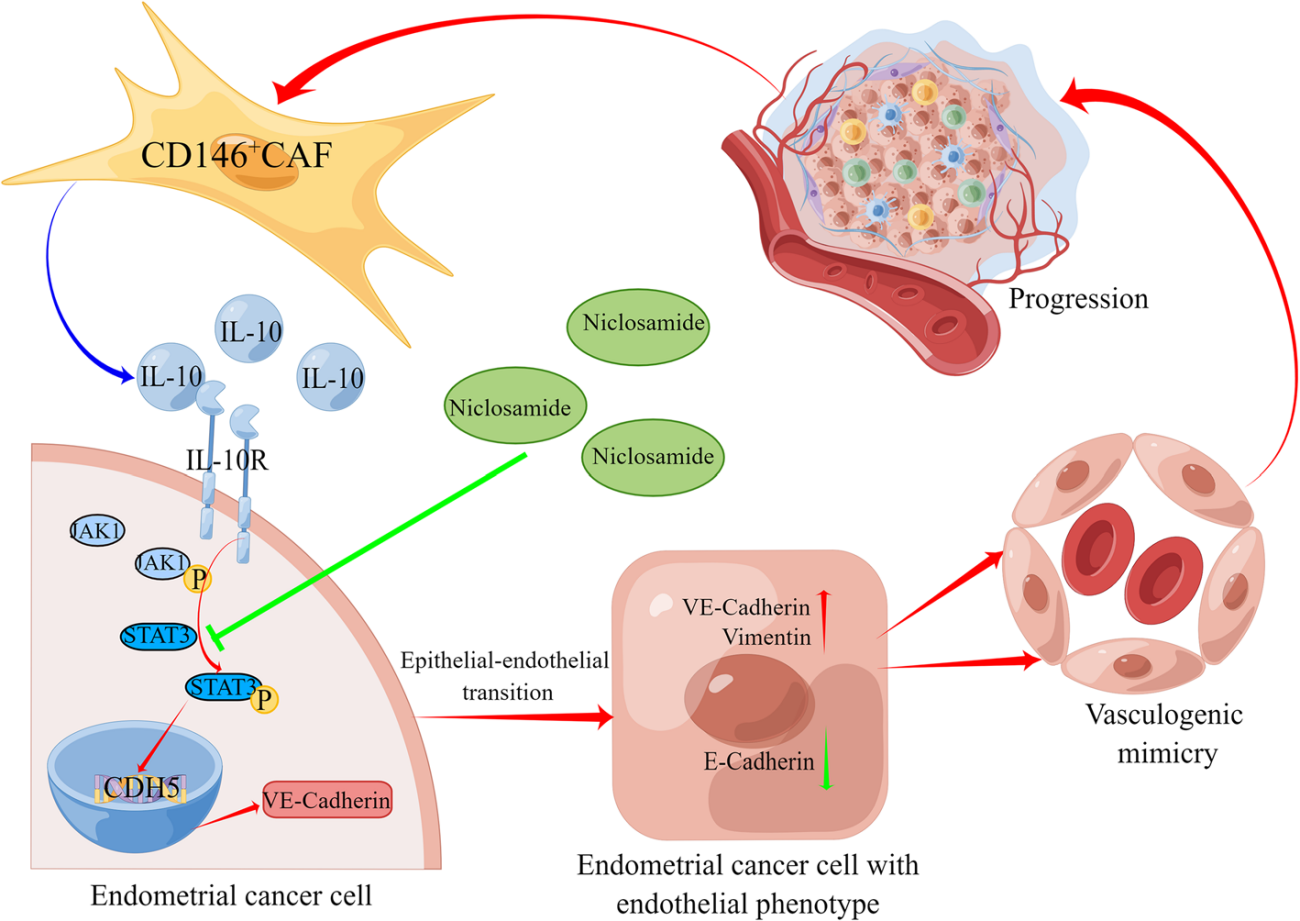
Schematic diagram of the mechanism of the full manuscript

We subsequently explored the role of CD146^+^CAFs in vivo. Coinjection of Ishikawa cells and CD146^+^CAFs significantly promoted the growth of endometrial cancer compared with injection of Ishikawa, Ishikawa + NF or Ishikawa + CD146^−^CAFs (Fig. 6A, B). The tumour weight of the Ishikawa + CD146^+^CAFs also increased markedly compared with that of the other tumours (Fig. 6C). In addition, PAS and CD31 immunohistochemical staining revealed that the numbers of blood vessels and VM were more in Ishikawa + CD146^+^CAFs group with increased protein expression of VE-Cadherin and Vimentin and decreased E-cadherin expression (Fig. 6D). Taken together, these results provide evidence that CD146^+^CAFs could promote the progression of endometrial cancer by inducing angiogenesis and VM formation via the IL-10/JAK1/STAT3 pathway (Fig. 7).

## Discussion

In this study, we demonstrated the existence of CD146^+^CAFs in endometrial cancer. These cells can facilitate angiogenesis and VM formation in vitro and in vivo, ultimately contributing to tumour progression in endometrial cancer. IL-10 derived from CD146^+^CAFs can promote VM via the JAK1/STAT3 signalling pathway.

As one of the important blood supply modes in the tumour microenvironment, VM has been reported in numerous tumours. In hepatocellular carcinoma (HCC), hypoxia-inducible factor-1α (HIF-1α) can promote VM formation through LOXL2 upregulation and eventually lead to the metastasis and progression of HCC [21]. The formation of VM was also associated with the overall survival (OS) of non-small cell lung cancer (NSCLC) patients. Thrombin can induce VM formation via PAR-1-mediated NF-κB signalling cascades, which provides potential therapeutic targets for NSCLC [22]. A systemic review of 22 clinical studies derived from data concerning VM and the 5-year survival of 3,062 patients across 15 cancer types showed that tumour VM is correlated with poor prognosis [23]. Therefore, it is perspective to carry out clinical trials targeting VM. Several anti-VM therapeutic agents such as dequalinium (DQA)-modified paclitaxel plus ligustrazine micelles, thalidomide, trastuzumab, tapamycin and resveratrol have been identified [24, 25]. The JAK1/STAT3 signalling pathway inhibitor niclosamide also inhibited VM in oral cancer [26]. In addition, drugs that simultaneously target VM and the endothelium can block the blood supply of glioma patients and inhibit the growth of tumours more completely and efficiently than each agent alone [27]. We have elucidated that niclosamide can inhibit endometrial cancer cells VM formation in vitro in this study. However, We lack validation in vivo experiments and clinical samples.

CAFs and their markers may be effective targets for antitumor therapy and drug design due to their stable genome [28]. In gallbladder cancer, CAFs can promote VM formation and tumour growth by upregulating NOX4 expression through activation of the IL-6-JAK-STAT3 signalling pathway [29]. CAFs play an important role in VM formation through various signalling pathways, such as EphA2-PI3K and ERK1/2 pathways, in gastric cancer [30, 31]. The conditioned medium of hepatocellular CAFs promoted VM formation and the expression of VM-related genes and proteins (MMP2 and EphA2) in hepatoma cell lines [32]. However, limited studies on VM in endometrial cancer have been reported. Our research provides new insights into VM formation in endometrial carcinoma. CD146 is not only an adhesion molecule (MCAM), but also a cellular surface receptor for miscellaneous ligands [33]. Numerous studies have revealed that CD146^+^CAFs can contribute to the progression of colorectal cancer (CRC) and intrahepatic cholangiocarcinoma (ICC) and might be potential targets for cancer therapy [34, 35]. However, the biologic effect could be influenced by numerous factors such as miRNAs and complicated signaling pathways [36, 37].

The relationship between IL-10 and angiogenesis is still controversial. IL-10-conditioned fibroblast media could significantly promote endothelial sprouting and network formation [38]. In endometriosis, IL-10 secreted from plasmacytoid dendritic cells could also promote angiogenesis in the early stage [39]. As an important factor for poor prognosis, the primary effect of IL-10 on lung cancer cells may be to increase their metastatic potential by promoting angiogenesis and resistance to apoptosis [40]. We also found that the expression of IL-10 is correlated with OS in endometrial cancer patients. However, IL-10 as a therapeutic target still requires extensive preclinical research. Some evidence has indicated that IL-10 has suppressive effects on angiogenesis, tumour growth, and peritoneal dissemination of VEGF-producing ovarian cancer cells [41]. Therefore, the specific function of IL-10 in cancer should be explored in further experiments.

Taken together, we demostrate that CD146^+^CAFs can promote progression of endometrial cancer by inducing angiogenesis and vasculogenic mimicry via IL-10/JAK1/STAT3 pathway. We have to admit that the limitation of this study is lack of clinical samples and in vivo experiments. But our findings may reveal a new molecular target for inhibiting angiogenesis and anti-VM formation, which lead to new treatment options for patients with advanced endometrial cancer.

### Supplementary Information

**Additional file 1: Figure S1.** IF of the primary isolated normal fibroblasts and cancer-associated fibroblasts. **Figure S2. **IL-10 is a risk factor for endometrial cancer. (A) The transcriptome level of IL-10 was correlated with MCAM; (B) The immunohistochemical staining of IL-10 in endometrial cancer and normal endometrium; (C) Prognosis significance of IL-10 in endometrial cancer by The Human Protein Atlas; (D) The expression level of IL-10 receptors in endometrial cancer cell lines; (E) The numbers of VM in IL-10 low expression and high expression endometrial cancers. **Figure S3. **IL-10 induces VM via JAK-STAT3 pathway. (A) The VM formation induced by IL-10 was blocked by Niclosamide; (B) The transcriptome level of CDH5 was correlated with JAK1 and STAT3; (C) The length of the qPCR product was tested by DNA gel.

## Data Availability

No datasets were generated or analysed during the current study.
