# Supplementary figures and images for: CD146+CAFs promote progression of endometrial cancer by inducing angiogenesis and vasculogenic mimicry via IL-10/JAK1/STAT3 pathway
Source: Cell Commun Signal. 2024 Mar 8;22:170. doi: 10.1186/s12964-024-01550-9 (PMC10921754; doi:10.1186/s12964-024-01550-9)

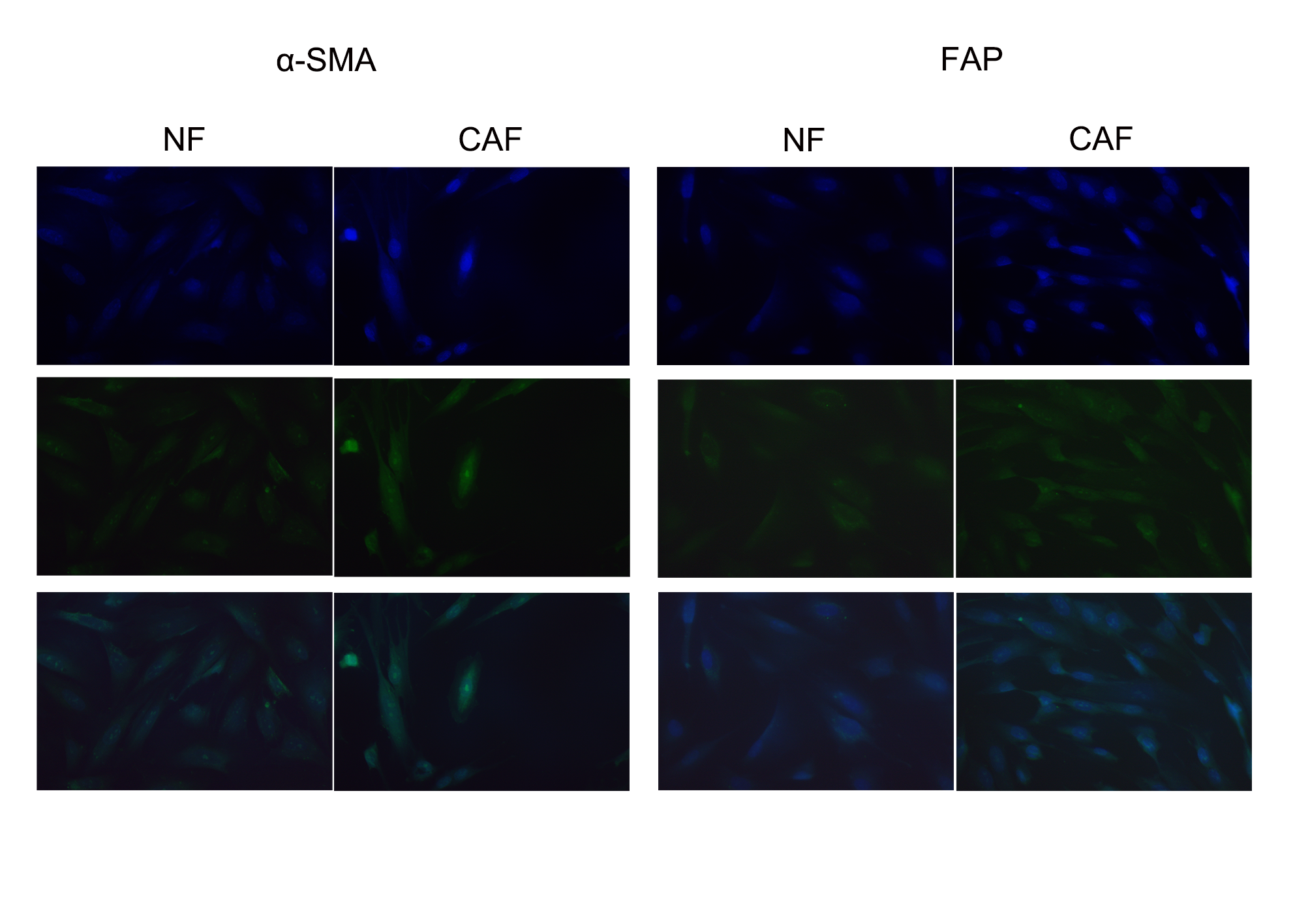

Supplement: Supplementary file 1 — Additional file 1: Figure S1. IF of the primary isolated normal fibroblasts and cancer-associated fibroblasts. Figure S2. IL-10 is a risk factor for endometrial cancer. (A) The transcriptome level of IL-10 was correlated with MCAM; (B) The immunohistochemical staining of IL-10 in endometrial cancer and normal endometrium; (C) Prognosis significance of IL-10 in endometrial cancer by The Human Protein Atlas; (D) The expression level of IL-10 receptors in endometrial cancer cell lines; (E) The numbers of VM in IL-10 low expression and high expression endometrial cancers. Figure S3. IL-10 induces VM via JAK-STAT3 pathway. (A) The VM formation induced by IL-10 was blocked by Niclosamide; (B) The transcriptome level of CDH5 was correlated with JAK1 and STAT3; (C) The length of the qPCR product was tested by DNA gel. [file 12964_2024_1550_MOESM1_ESM.zip › Figure S1.tif]

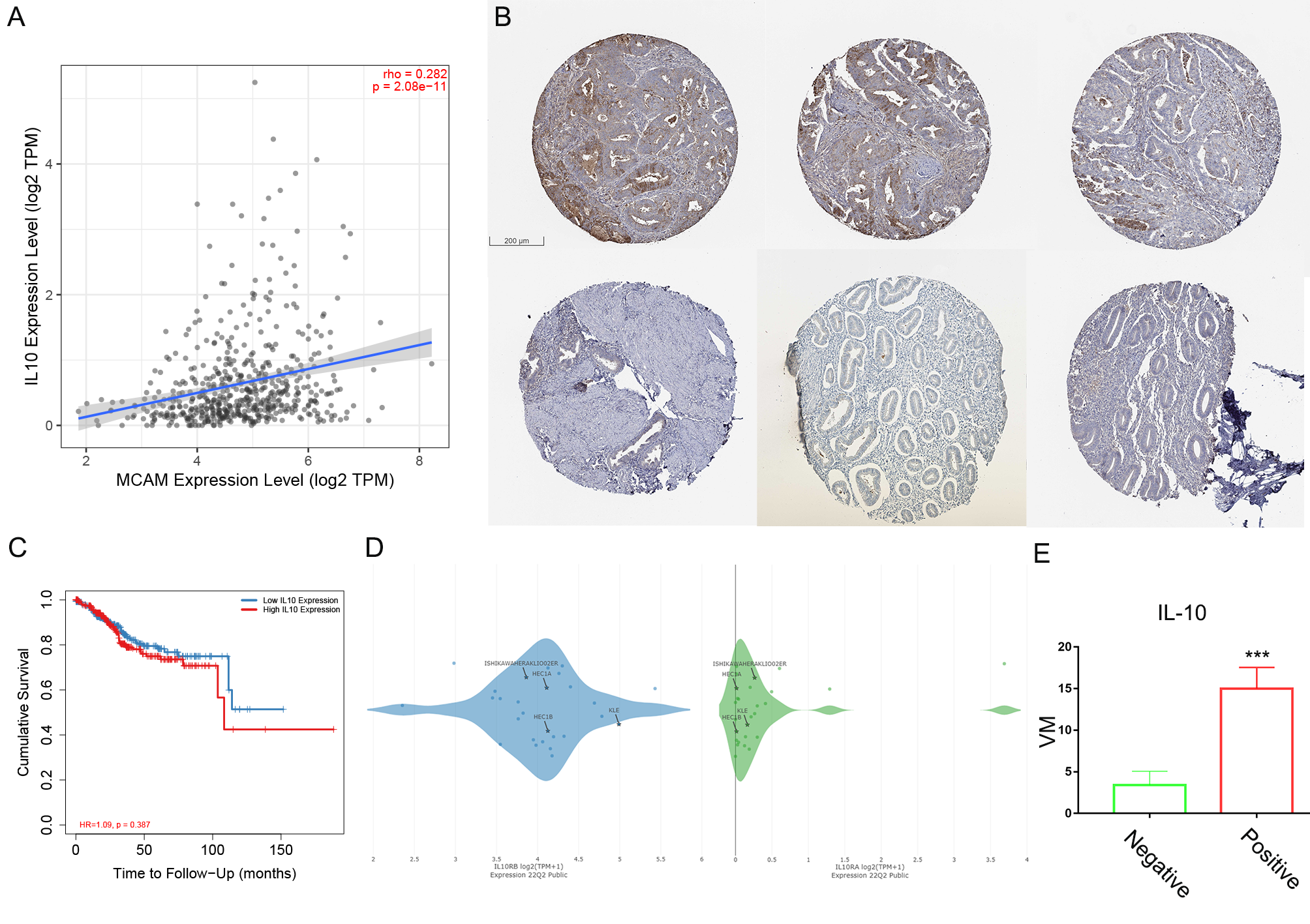

Supplement: Supplementary file 1 — Additional file 1: Figure S1. IF of the primary isolated normal fibroblasts and cancer-associated fibroblasts. Figure S2. IL-10 is a risk factor for endometrial cancer. (A) The transcriptome level of IL-10 was correlated with MCAM; (B) The immunohistochemical staining of IL-10 in endometrial cancer and normal endometrium; (C) Prognosis significance of IL-10 in endometrial cancer by The Human Protein Atlas; (D) The expression level of IL-10 receptors in endometrial cancer cell lines; (E) The numbers of VM in IL-10 low expression and high expression endometrial cancers. Figure S3. IL-10 induces VM via JAK-STAT3 pathway. (A) The VM formation induced by IL-10 was blocked by Niclosamide; (B) The transcriptome level of CDH5 was correlated with JAK1 and STAT3; (C) The length of the qPCR product was tested by DNA gel. [file 12964_2024_1550_MOESM1_ESM.zip › Figure S2.tif]

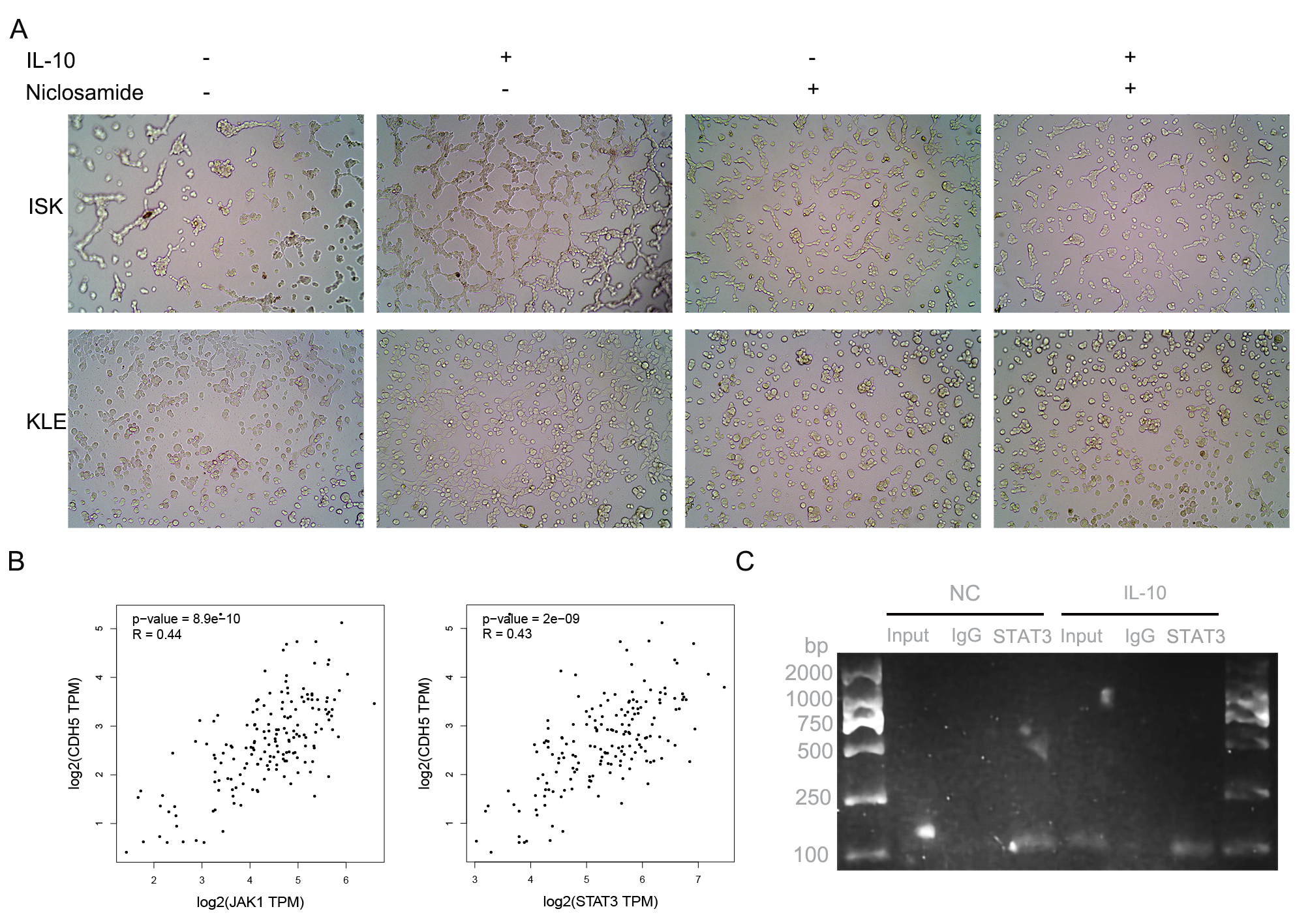

Supplement: Supplementary file 1 — Additional file 1: Figure S1. IF of the primary isolated normal fibroblasts and cancer-associated fibroblasts. Figure S2. IL-10 is a risk factor for endometrial cancer. (A) The transcriptome level of IL-10 was correlated with MCAM; (B) The immunohistochemical staining of IL-10 in endometrial cancer and normal endometrium; (C) Prognosis significance of IL-10 in endometrial cancer by The Human Protein Atlas; (D) The expression level of IL-10 receptors in endometrial cancer cell lines; (E) The numbers of VM in IL-10 low expression and high expression endometrial cancers. Figure S3. IL-10 induces VM via JAK-STAT3 pathway. (A) The VM formation induced by IL-10 was blocked by Niclosamide; (B) The transcriptome level of CDH5 was correlated with JAK1 and STAT3; (C) The length of the qPCR product was tested by DNA gel. [file 12964_2024_1550_MOESM1_ESM.zip › Figure S3.tif]

## Full uncut gels

**Figure 2E**

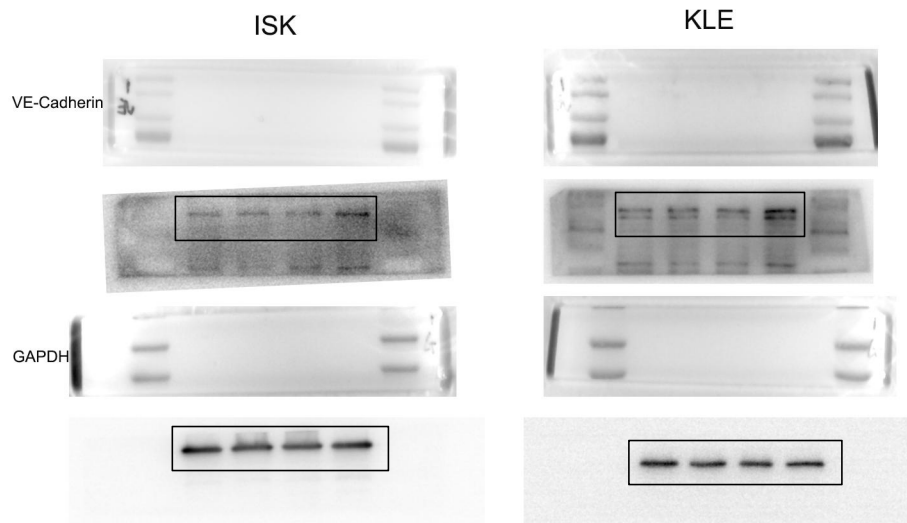

**Figure 3B**

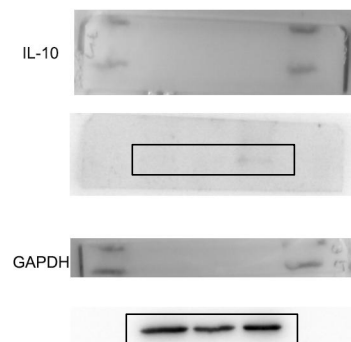

Figure 3E

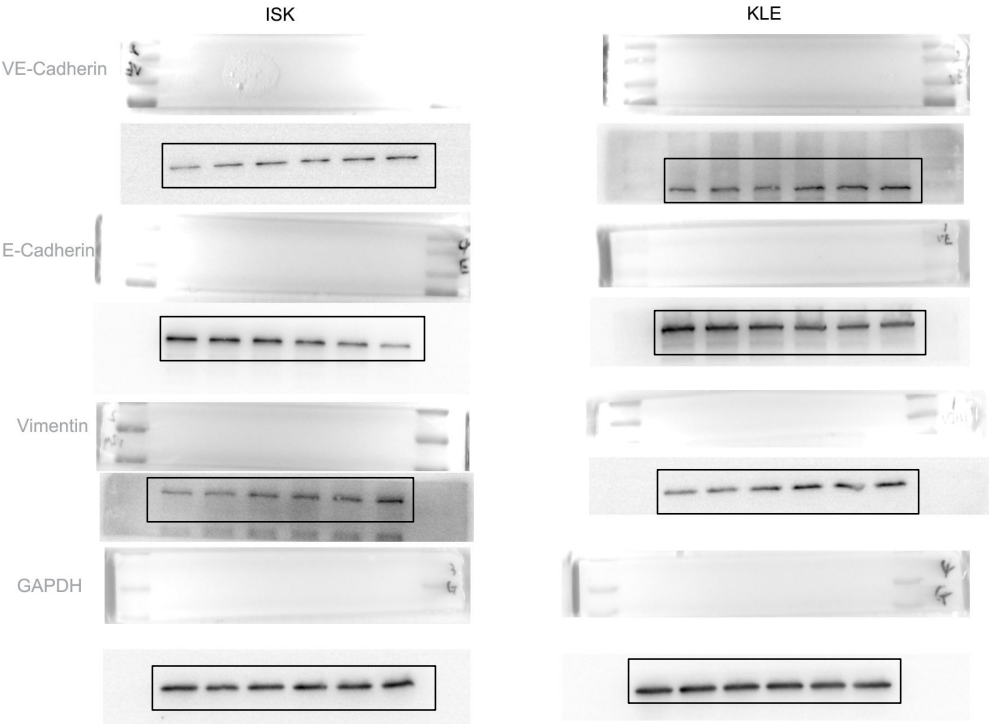

Figure 5B

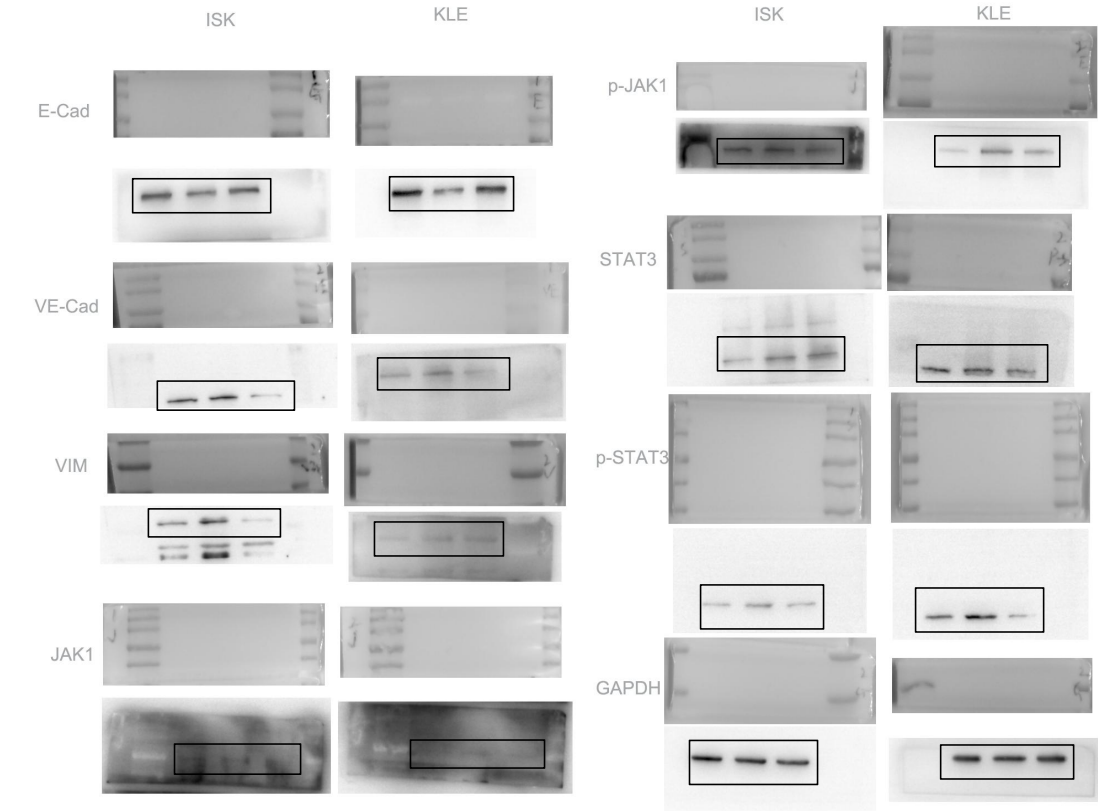

Figure 5E

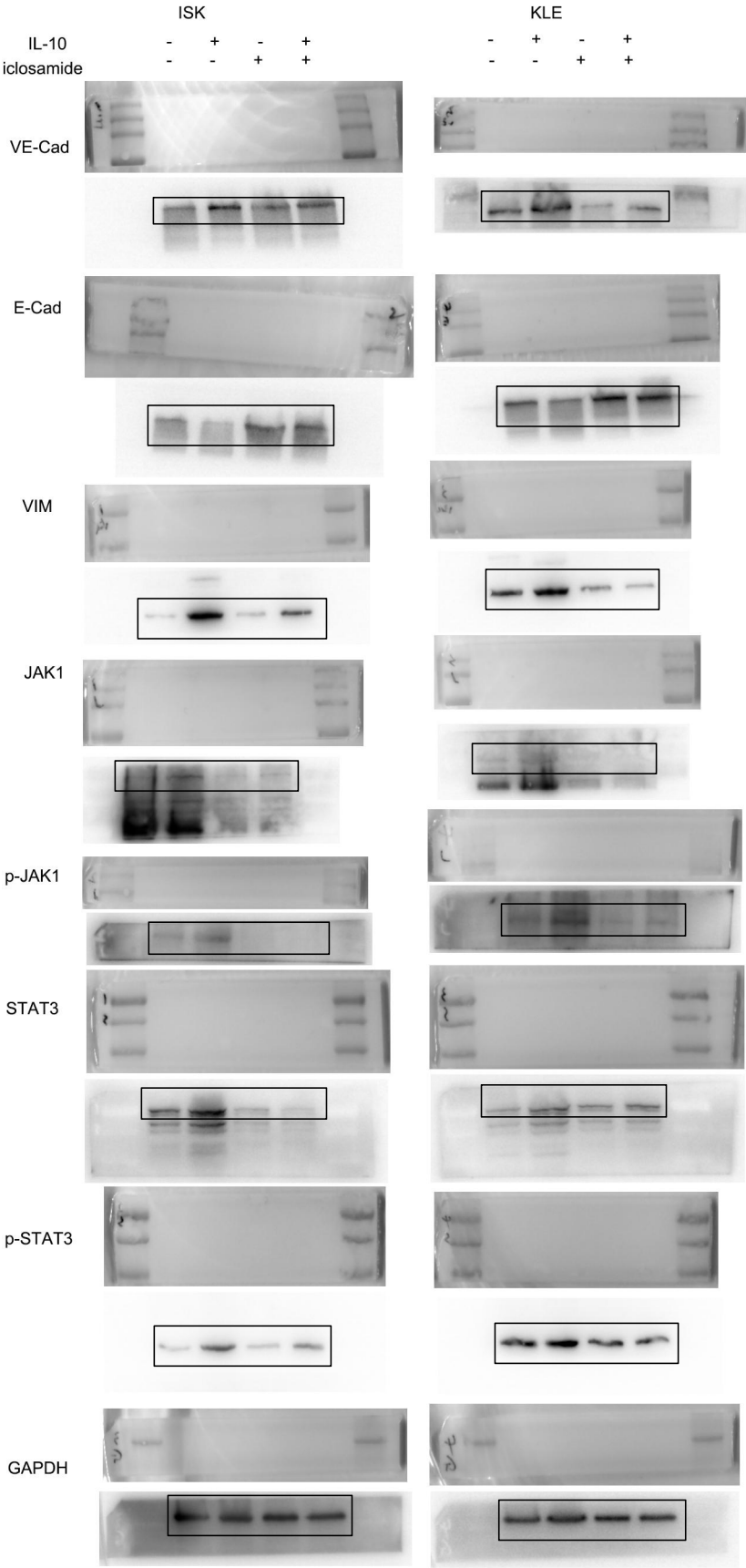

Supplement: Supplementary file 1 — Additional file 1: Figure S1. IF of the primary isolated normal fibroblasts and cancer-associated fibroblasts. Figure S2. IL-10 is a risk factor for endometrial cancer. (A) The transcriptome level of IL-10 was correlated with MCAM; (B) The immunohistochemical staining of IL-10 in endometrial cancer and normal endometrium; (C) Prognosis significance of IL-10 in endometrial cancer by The Human Protein Atlas; (D) The expression level of IL-10 receptors in endometrial cancer cell lines; (E) The numbers of VM in IL-10 low expression and high expression endometrial cancers. Figure S3. IL-10 induces VM via JAK-STAT3 pathway. (A) The VM formation induced by IL-10 was blocked by Niclosamide; (B) The transcriptome level of CDH5 was correlated with JAK1 and STAT3; (C) The length of the qPCR product was tested by DNA gel. [file 12964_2024_1550_MOESM1_ESM.zip › Full uncut gels.pdf]
